# Effects of Channel Characteristics on Wastewater Chemical Transformation in Rivers

**DOI:** 10.1007/s11270-026-09211-y

**Published:** 2026-02-05

**Authors:** Robert A. Newbould, D. Mark Powell, Juliet Hodges, Alexandre Teixeira, Michael J. Whelan

**Affiliations:** 1 School of Geography, Geology and the Environment, University of Leicester, Leicester, LE1 7RH UK; 2 Unilever Safety, Environmental and Regulatory Science (SERS), Colworth Science Park, Sharnbrook, MK44 1LQ UK; 3 Present Address: Yorkshire Water Services, Bradford, BD6 2SZ UK

**Keywords:** Geomorphology, Sewage, Mesocosm, Ammonia, Linear alkylbenzene sulphonate, Surfactants

## Abstract

Microbially-mediated transformations are important for removing wastewater chemicals in rivers. In-stream degradation rates are likely to be affected by channel morphology and size because these factors determine the extent of chemical contact between the water column and the microbial community in fixed biofilms, where most microbially-mediated processes are expected to take place. We hypothesize that transformation rate constants are inversely proportional to hydraulic radius (*R*: the ratio of channel cross-sectional area to wetted perimeter). Microbial transformations are also predicted to be controlled systematically by bed sediment characteristics. This will be a function of sediment surface area per unit volume (for biofilm colonisation) and hyporheic exchange. Here, we test the above hypotheses in controlled 14-day laboratory experiments, by monitoring nitrification and biodegradation in stirred tanks with three different water depths and three different sediment sizes (sand, gravel and cobbles). Transformation rate constants were inversely proportional to depth, supporting the notion that channel morphology represents a significant system-specific control. Rate constants for nitrification were highest in the gravel treatments and lowest in sand. This suggests that intermediate grain sizes simultaneously allow good biofilm colonisation and solute exchange between sediment and the water column. There was, however, little difference in biodegradation rate constants between different sediment sizes. This study highlights the need to consider geomorphology in models of chemical exposure.

**Supplementary Information:**

The online version contains supplementary material available at 10.1007/s11270-026-09211-y.

## Introduction

The emission of chemicals into surface waters via wastewater poses a potential risk for aquatic organisms. These include, *inter alia,* organic chemicals, such as those found in pharmaceuticals and household products, and inorganic compounds, such as ammonia and heavy metals (Whelan et al., 2022). To calculate the concentration of chemicals of concern downstream of emission points, we need to understand how they dissipate in receiving environments under different conditions. In rivers, a number of mechanisms contribute to the removal of a chemical, including microbially-mediated transformations (e.g. nitrification and biodegradation), sorption to sediment, volatilisation, photodegradation and hydrolysis (Kunkel & Radke, 2011; Li et al., 2015; Zou et al., 2015). These mechanisms, and their relative importance, are affected by intrinsic properties of individual substances (e.g. partition coefficients and chemical structure) and environmental conditions, such as temperature, pH and short-wave radiation flux density.

For microbially-mediated transformations, contaminant behaviour will also be affected by the location and competence of microbial communities, including suspended colonies and fixed biofilms (Boeije et al., 2000; Kunkel & Radke, 2011; Li & McLachlan, 2019; Radke & Maier, 2014; Zou et al., 2015). These factors explain, in part, the wide range of biodegradation half-lives which have been reported for organic contaminants in different environmental systems (Boethling et al., 2009). Fixed biofilms (also known as the periphyton) are believed to be much more significant for the processing of chemicals in most rivers and streams, compared to suspended organisms, because their biomass is typically higher and their communities are more diverse (Boeije et al., 2000; Exton et al., 2023; Honti et al., 2018; Takada et al., 1994). It is for this reason that sediments at the sediment–water interface and in the hyporheic zone are often regarded as ‘bioreactors’, with high potential to degrade wastewater contaminants (Battin et al., 2016; Lewandowski et al., 2011; Roche et al., 2019).

The high importance of biofilms at the sediment–water interface means that microbially-mediated transformations should be affected by contact between the water column and sediment surfaces. This is controlled to some extent by channel shape and size (Alexander et al., 2000; Honti et al., 2018; Liu et al., 2020). Channels with a high interaction between the water column and sediment surfaces (i.e. shallow rivers and streams) will have a low hydraulic radius (*R*: the ratio of channel cross-sectional area to wetted perimeter). We hypothesise that rate constants for microbially-mediated transformations (*k*) will be inversely proportional to *R* (Boeije et al., 2000; Price et al., 2009):

1 $$k \propto \frac{1}{R}$$

In wide rivers, hydraulic radius is approximately equal to mean depth (Leopold & Maddock, 1953).

Similar explanations have been invoked for observations of reduced nitrification rates with increasing depth in rivers and free surface constructed wetlands (Al-Lami et al., 2021; Pauer & Auer, 2009) and for denitrification in lakes, reservoirs and rivers (Alexander et al., 2000; Howarth et al., 1996; Kelly et al., 1987; Seitzinger et al., 2002; Smith et al., 1997). For example, Alexander et al. (2000) and Böhlke et al. (2009) found substantial reductions in in-stream losses of nitrogen in the Mississippi river with increasing channel depth, implying that pollutant inputs in lower reaches are more likely to be exported to the ocean that inputs from the headwaters. Similar concepts have also been explored for chemical biodegradation in rivers (e.g. Boeije et al., 2000; Honti et al., 2018), but the evidence base is smaller and the notion that rate constants change systematically with channel dimensions is not routinely considered in catchment-scale models of chemical exposure, such as GREAT-ER (Feijtel et al., 1997; Koormann et al., 2006; Lämmchen et al., 2021).

Microbially-mediated degradation should also be affected by characteristics of the bed sediment. Specifically, the sediment surface area (SSA) will control the space available for biofilm colonisation. SSA per unit volume of bed material increases with decreasing particle size, so microbially-mediated degradation is expected to be more rapid in fine-bedded streams, compared to coarse-bedded streams (Cook et al., 2020; Parker et al., 2018). This hypothesis is supported by Parker et al. (2018) who measured biofilm community metabolism and nutrient uptake in differently-sized sediment treatments in a stream microcosm experiment. They found a significant decline in rates of biogeochemical function (biofilm community respiration and uptake of ammonium and phosphate) with increasing particle size. However, the relationship between sediment particle size and microbially-mediated transformations in river channels is likely to be complicated by other factors – particularly hyporheic exchange, which will regulate the transport of solutes to biofilm communities below the sediment–water interface. For example, the permeability of fine sediment is typically low, which inhibits the advection of nutrients for biofilm development at depth and the penetration of chemicals for processing (Cook et al., 2020; O’Connor & Harvey, 2008). Furthermore, the ‘clogging’ of interstitial pore spaces due to biofilm development or the deposition of fine particles can lead to a reduction in hydraulic conductivity which could confound simple relationships between primary particle size and transformation rate constants (Aubeneau et al., 2016; Muñoz-Vega et al., 2023; Nogaro et al., 2010). This phenomenon is anticipated to exert a greater impact on fine and intermediate sediment sizes (such as silt and sand) compared to coarse sediment sizes (such as gravels and cobbles) because pore diameters are lower and, thus, more susceptible to infilling.

Surprisingly little research has been conducted on the effects of channel geometry and sediment calibre on the transformation of wastewater chemicals. There are experiments investigating other variables, such as flow velocity (Kunkel & Radke, 2008) and the effects of bedform (Li et al., 2015; Posselt et al., 2020), as well as research on the effect of grain size on hyporheic exchange (Chandler et al., 2016; Cook et al., 2020; Nagaoka & Ohgaki, 1990), but the latter rarely relate differences in exchange to microbially-mediated degradation rates. To date there are few, if any, empirical studies which explicitly investigate the combined effects of depth and sediment calibre on biotransformation rates. Here, we test the hypotheses that microbially-mediated rate coefficients will be inversely proportional to (1) hydraulic radius and (2) sediment particle size, in deliberately simplified and controlled laboratory experiments, in which nitrification and biodegradation were monitored in tanks with different depth and sediment size treatments. The experiments used river water spiked with high concentrations of ammoniacal nitrogen and linear alkylbenzene sulphonate (LAS), both of which are commonly released to rivers and streams via wastewater. The experiments were designed to test the variables of interest, but were not designed to be fully representative of the natural environment.

## Methods

### Apparatus

**Fig. 1:**
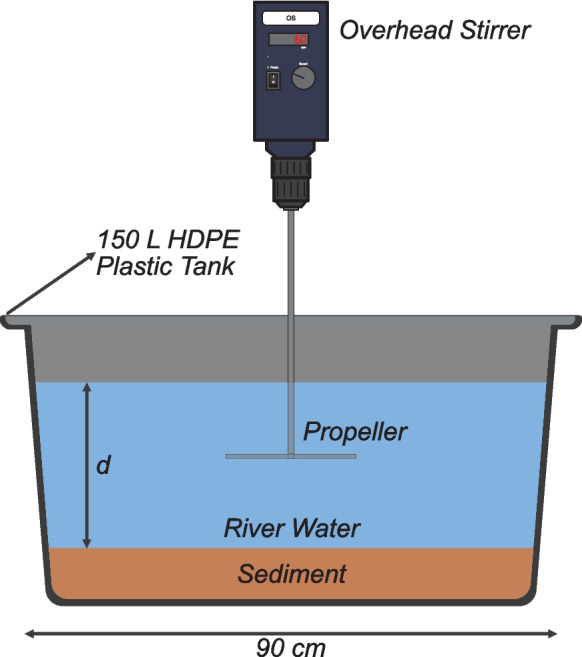
Schematic of the experimental design. *d* is depth above the sediment–water interface

Two experiments were set up investigating the influence of: (1) channel geometry and (2) sediment size, on nitrification and biodegradation. In all cases, river water was added to cylindrical 150 L HDPE plastic tanks containing well-sorted commercially-sourced sediment (Tarmac, Solihull, UK). A slow-moving overhead stirrer (~ 60 rpm) was used to generate turbulence and promote mixing (Fig. 1). The stirrer’s propeller was placed in the centre of each tank at 0.5 *d*, where *d* is depth above the sediment–water interface. Nine tanks were set up in each experiment, which allowed for three replicates of three treatments per experiment.

In the channel geometry experiment, depth was altered by varying the volume of water added to each tank. The treatments were: (1) low depth (*d* = 4 cm) – 20 L water; (2) medium depth (*d* = 11 cm) – 50 L water; and (3) high depth (*d* = 24.5 cm) – 100 L water. 10 kg of gravel (*D* ≈ 5 mm, where *D* is sediment size) was placed in each tank. It was assumed that biofilm would form predominantly on the sediment, where the surface area was high, with minimal growth expected on the tank walls (i.e. wetted perimeter was assumed equal to width and hydraulic radius was assumed equal to depth).

In the sediment size experiment, the treatments were: (1) sand (*D* ≈ 0.25 mm, *ρ*_*b*_ ≈ 1648 kg m^−3^, *ϕ* ≈ 38%, where *ρ*_*b*_ is bulk density and *ϕ* is porosity), (2) gravel (*D* ≈ 5 mm, *ρ*_*b*_ ≈ 1583 kg m^−3^, *ϕ* ≈ 40%), and (3) cobbles (*D* ≈ 50 mm, *ρ*_*b*_ ≈ 1561 kg m^−3^, *ϕ* ≈ 41%). The depth of the sediment layer was 5 cm in each treatment. This equated to 23 kg sand, 30 kg gravel and 35 kg cobbles. 50 L of river water (*d* = 11 cm) was added to each tank.

The water level in each tank in each experiment was monitored for evaporative losses of water, which were replaced. Each experiment was conducted under low-light conditions to reduce the proliferation of phototrophs and minimise any potential chemical removal through photodegradation, although both test chemicals are known to be photostable.

### River Water and Biofilm Development

River water was collected from the River Sence (a tributary of the River Soar), Wistow, Leicestershire, UK (52.562787°N, 1.056119°W) and added to each tank < 3 h after collection. Land cover in the Sence catchment is dominated by arable farmland (42%) and grazed/cut grassland (41%: UK National River Flow Archive). The catchment also receives multiple inputs of treated sewage effluent. It was assumed, given the nutrient intensive land use and wastewater inputs in the catchment, that sufficient nutrients for biofilm growth were present in the river water. Further information about the study area can be found in Newbould et al. (2021). After the river water was added, each tank was left for three weeks to allow a biofilm to develop. This period was based on a preliminary experiment in which biofilm development was measured on extractable glass slides placed on the sediment surface (Cook et al., 2020; Kowalczyk et al., 2016). Extracellular polysaccharides were measured using a glucose standard as a proxy for biofilm biomass via a phenol–sulfuric acid assay (DuBois et al., 1956; Liu et al., 1994). Biofilm growth was initially rapid, but reached a steady-state before day 21. The three-week acclimation period is also consistent with that employed in other work (Cook et al., 2020; Kowalczyk et al., 2016; Li et al., 2015). Although microbial transformation rates are influenced by biofilm community composition and function (Davenport et al., 2022; Kowalczyk et al., 2015, 2016), we did not characterise the biofilm in each experiment. It was assumed that biofilm characteristics would be similar between treatments in each experiment because the river water was collected from the same location and at the same time.

### Nitrification and Biodegradation Monitoring

After acclimation, the river water in each tank was spiked with ammonium sulphate, (NH_4_)_2_SO_4_, and sodium dodecylbenzene sulphonate, SDBS, to nominal concentrations of 15 mg N L^−1^ and 2 mg L^−1^, respectively. SDBS is a common ingredient of laundry detergents, more commonly referred to as LAS. LAS is a multi-constituent substance composed of alkyl chain homologues of varying lengths (C_10_ to C_13_) and positional isomers (Fig. S1; Table S1). SDBS is essentially the C_12_ LAS homologue. Note that 2 mg L^−1^ of SDBS is equivalent to 1.55 mg L^−1^ of LAS, after accounting for the sodium content (6.6%) and purity (83%) of SDBS. These concentrations are relatively high to ensure they could be easily measured and their degradation observed, but are within the range previously reported downstream of sewage treatment plant effluents under low dilution and under untreated household wastewater conditions (Boeije et al., 2000; Finnegan et al., 2009). Both chemicals were selected due to their widespread presence in wastewater and due to their degradation profiles which are heavily influenced by the presence of biofilms (Boeije et al., 2000; Menzies et al., 2017; Pauer & Auer, 2009). (NH_4_)_2_SO_4_ (molecular biology grade, ≥ 99.0%) and SDBS (technical grade, 83% purity) were supplied by Sigma-Aldrich (Gillingham, UK). The nitrification of ammonium (NH_4_^+^) and biodegradation of LAS were monitored over a two-week period by taking three replicate samples of 4 mL each, at least 3 times per week. Water samples were filtered through 0.45 μm PTFE syringe filters prior to any analyses. Temperature, pH and dissolved oxygen (DO) concentrations were also measured before each sample was taken.

NH_4_^+^-N concentrations were determined colorimetrically on the day of collection with a method equivalent to ISO 15923–1 using a SEAL AQ2 discrete analyser. All reagents used were ACS reagent grade (≥ 95%) or better. Nitrification was quantified through NH_4_^+^-N depletion over time. The measurement of NO_3_^—^N was not performed due to intermittent instrument issues. LAS concentrations were determined using liquid chromatography with tandem mass spectrometry (Agilent 1290 series LC–MS/MS system with 6495 triple quadrupole mass spectrometer) against a LAS standard (97.3%) supplied by Cepsa (Madrid, Spain). Water samples analysed with LC–MS/MS were frozen on the day of collection and analysed in batches, against standards prepared in ultra-pure water. Further details on the LC–MS/MS conditions, as well as information on method development and validation, are available in the Supplementary Materials.

During this part of the experiment, evaporative losses of water were topped up with deionized water to prevent changes due to evaporative concentration and to prevent new sources of NH_4_^+^ and LAS entering the system. There was a small dilution effect when the water depth was topped up to its original level because only part of the replacement volume was for evaporation and the rest was to replace water removed via sampling. However, this dilution effect was minimal at the high-water volumes used in these experiments. For example, the volume of water removed via sampling represents < 0.5% of the total water volume used in the low-depth treatment – even less in the medium and high-depth treatments.

### Modelling

Trends in NH_4_^+^-N and LAS concentrations can be described by zero-order (ZO) or first-order (FO) degradation kinetics with the incorporation of a lag period. Although FO kinetics often apply (Chapra, 2008; Le et al., 2019), ZO kinetics have been observed previously for nitrification (Pauer & Auer, 2000; Whelan et al., 2007). The lag period between the start of the experiment and the onset of degradation is typically caused by delayed growth of a competent organism in the microbial consortium (Le et al., 2019; Strotmann et al., 2023). This was described, for both nitrification and biodegradation, with a Gompertz function of the form:

2 $${m}_{t}= {e}^{-b{e}^{-ct}}$$

where ${m}_{t}$ is the relative size of the competent microbial population (0–1) at time $t$ (h) and $b$ and $c$ are parameters, which are calibrated as part of the optimization procedure (described below) (Zwietering et al., 1990). The Gompertz function has a sigmoid form in which microbial growth is initially slow, followed by an exponential phase and then a stationary phase. This leads to Eqs. 3 and 4 for ZO and FO degradation kinetics, respectively:

3 $${C}_{t}=\overline{{C }_{max}}-\left(k . {m}_{t }. t\right)$$

4 $${C}_{t}= \overline{{C }_{max}} . {e}^{(-k { . m}_{t} . t)}$$

where ${C}_{t}$ is concentration at time $t$ (mg N L^−1^ for NH_4_^+^ and μg L^−1^ for LAS), $\overline{{C }_{max}}$ is the peak concentration averaged across all treatments and $k$ is a rate constant with units of mg L^−1^ h^−1^ for zero order kinetics and h^−1^ for first order kinetics.

To calibrate each model, $b$, $c$ and $k$ were estimated by iterative optimisation to reduce the root-mean-square error (RMSE) between observed and modelled concentrations using the generalized reduced gradient algorithm (Lasdon et al., 1978). In some cases, observed concentrations of NH_4_^+^ and LAS were below the limits of detection before the end of the experiment. To accurately describe ZO transformation kinetics, the first observed concentration < 20% of $\overline{{C }_{max}}$ was included in the optimization of ZO transformation curves, but subsequent concentrations were not. This avoided unrealistically high predicted concentrations in the later stages of the experiment.

Some degree of equifinality was observed during calibration, in which different parameter combinations resulted in similar model performance (Beven & Freer, 2001; von Bertalanffy, 1968; Whelan et al., 2019). For example, unconstrained optimisation resulted in unrealistically high values for $k$ because predicted microbial growth was low (${m}_{t}$ ≪ 1) for the whole experimental period. To overcome this problem, an additional constraint was applied to ensure that ${m}_{t}$ was ≥ 0.95 on day 7 (i.e. assuming that a competent microbial population was well-developed on or before day 7). This is consistent with other work where the lag period for ammonium oxidation was observed to be between 0 and 7 days (Cavari, 1977; Le et al., 2019; Pauer & Auer, 2000; Xia et al., 2004; Yongming, 1988). Seven days was also considered to be an appropriate maximum lag period for the biodegradation of LAS, based on short lag periods observed in biodegradation simulation tests (Federle & Itrich, 1997; Larson, 1990; León et al., 2004; Peng et al., 2000). Note that Eq. 2 approaches an asymptote close to but never equal to unity.

### Statistical Analyses

A ‘bootstrapping’ approach was used to derive confidence intervals for $k$ in each model, experiment and treatment empirically (Effron & Tibshirani, 1993). This involved randomly sampling (with replacement) nine measured concentrations of NH_4_^+^ or LAS for each sampling point and fitting an optimum value for $k$ based on the average concentration. The optimum value for $k$ was determined by minimising the RMSE between measured and modelled concentrations using the BFGS (Broyden-Fletcher-Goldfarb-Shanno) algorithm (Fletcher, 2000). Note that the BFGS algorithm was not used for initial optimization because it is not designed to handle problems with additional constraints (i.e. that microbially growth must be well-developed by day 7). The bootstrapping process was iterated 1000 times to derive a distribution of values for $k$. A Kruskal–Wallis test was then conducted on the distribution of $k$-values to determine if there was a significant difference in $k$ between treatments.

## Results and Discussion

### Channel Geometry Experiment

**Fig. 2:**
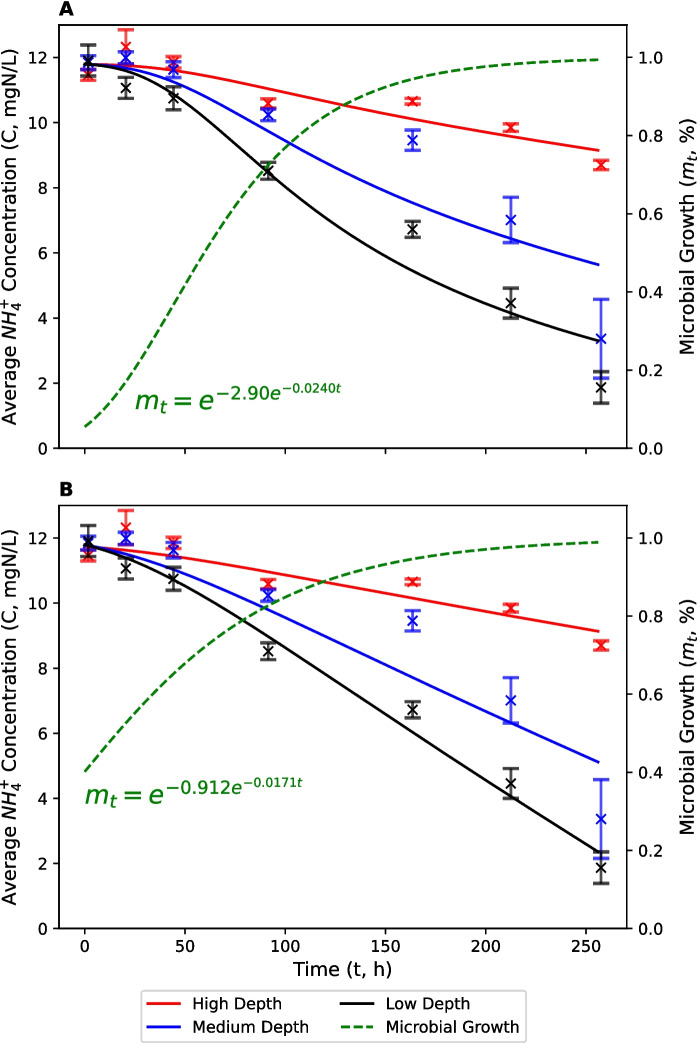
Average concentrations of NH_4_^+^-N in low (black), medium (blue) and high-depth (red) treatments. Solid lines represent modified first-order (**A**) and zero-order (**B**) nitrification models which account for a lag period. The calibrated microbial growth curve (green/dashed) is also shown. Error bars represent standard errors

The average concentration of NH_4_^+^ across all treatments at the start of the channel geometry experiment was 11.74 mg N L^−1^ (Fig. 2). This represents a small reduction in the nominal spiked concentration (15 mg N L^−1^), possibly caused by poor mixing, retention during filtration, sorption to sediment and volatilisation. This reduction was considered acceptable because it is small and consistent across treatments. NH_4_^+^ concentrations began to decrease after 43 h (~ 2 d). We assume that this represents the lag period for the development of nitrifying bacteria. The calibrated microbial growth curves were slightly different in the modified FO (Fig. 2a) and ZO (Fig. 2b) degradation models, with slightly faster calibrated microbial growth in the ZO model. In the absence of measured data on the relative abundance of nitrifying bacteria, it is not possible to determine which growth curve is most appropriate (Chao et al., 1993). Nitrification was quickest in the low-depth treatment, followed by the medium and high-depth treatments. Average NH_4_^+^ concentrations at the end of the experiment were 1.87, 3.36 and 8.7 mg N L^−1^, respectively. FO degradation rate constants ($k$) were 5.00 × 10^–3^, 2.90 × 10^–3^ and 9.97 × 10^–4^ h^−1^, which represent half-lives of 139, 239 and 695 h (Table 1). Fitted ZO degradation rate constants ($k$) were 3.73 × 10^–2^, 2.64 × 10^–2^ and 1.03 × 10^–2^ mg N L^−1^ h^−1^, respectively.

**Table 1:** Root-mean-square error (RMSE) and nitrification rate constants ($k$) for modified first-order and zero-order kinetics in the channel geometry experiment

	First-Order			Zero-Order		
Depth	$k$ (h^−1^)	RMSE (mg N L^−1^)	R^2^	$k$ (mg N L^−1^ h^−1^)	RMSE (mg N L^−1^)	R^2^
Low	5.00 × 10^–3^	0.755	0.95	3.73 × 10^–2^	0.408	0.99
Medium	2.90 × 10^–3^	1.16	0.86	2.64 × 10^–2^	1.01	0.91
High	9.97 × 10^–4^	0.423	0.87	1.03 × 10^–2^	0.425	0.91

**Fig. 3:**
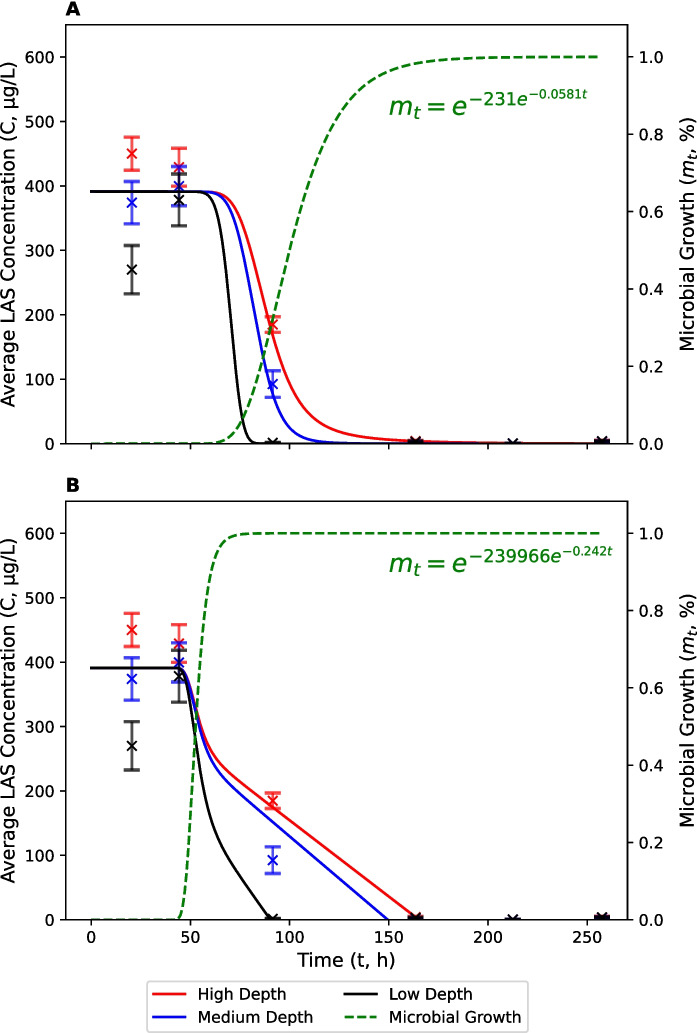
Average concentrations of LAS in low (black), medium (blue) and high-depth (red) treatments. Solid lines represent modified first-order (**A**) and zero-order (**B**) biodegradation models which account for a lag period. The calibrated microbial growth curve (green/dashed) is also shown. Error bars represent standard errors

The average concentration of LAS at the start of the channel geometry experiment ranged from 166 to 445 μg L^−1^. This represents a substantial reduction from the nominal spiked concentration (1.55 mg L^−1^). We believe that this was caused, at least in part, by a combination of matrix effects, which were calculated to account for a 35% reduction in spiked recoveries, and the sorption of LAS to sediment (see Supplementary Materials). The fact that average LAS concentrations across all treatments increased from 282 μg L^−1^ on day 0 (0 h) to 358 μg L^−1^ on day 1 (19 h) may have been due to mixing issues in the spiked solutions which were close to the limit of aqueous solubility for LAS (0.8 g L^−1^). There was also high variability between replicates in the medium-depth treatment at the start of the experiment (SEM = 192 μg L^−1^). Therefore, initial LAS concentrations were excluded from the optimization of FO and ZO biodegradation models (Fig. 3). Between day 1 (19 h) and day 4 (90 h), LAS concentrations decreased to 1, 93 and 185 μg L^−1^ in the low, medium and high-depth treatments, respectively. The lag period for LAS was similar to that for NH_4_^+^. Note that calibrated microbial growth curves are different in Figs. 2 and 3, but these differences are expected since NH_4_^+^ and LAS are transformed by different organisms (Goodnow & Harrison, 1972; Koops et al., 2006). Concentrations of LAS were below the limits of quantification (< LOQ) by 162 h in all treatments. Biodegradation followed the same trends as nitrification, with fastest rates observed in the low-depth treatment followed by the medium-depth and then high-depth treatments. FO degradation rate constants ($k$) were 4.78 × 10^–1^, 5.51 × 10^–2^ and 2.86 × 10^–2^ h^−1^, respectively (Table 2). These represent half-lives of 1.45, 12.6 and 24.2 h. ZO degradation rate constants ($k$) were 4.33, 2.62 and 2.37 μg L^−1^ h^−1^, respectively.

**Table 2:** Root-mean-square error (RMSE) and biodegradation rate constants ($k$) for modified first-order and zero-order kinetics in the channel geometry experiment

	First-Order			Zero-Order		
Depth	$k$ (h^−1^)	RMSE (μg L^−1^)	R^2^	$k$ (μg L^−1^ h^−1^)	RMSE (μg L^−1^)	R^2^
Low	4.78 × 10^–1^	72.7	0.96	4.33	72.7	0.93
Medium	5.51 × 10^–2^	12.5	0.99	2.62	38.2	0.96
High	2.86 × 10^–2^	26.8	0.99	2.37	27.1	0.99

**Fig. 4:**
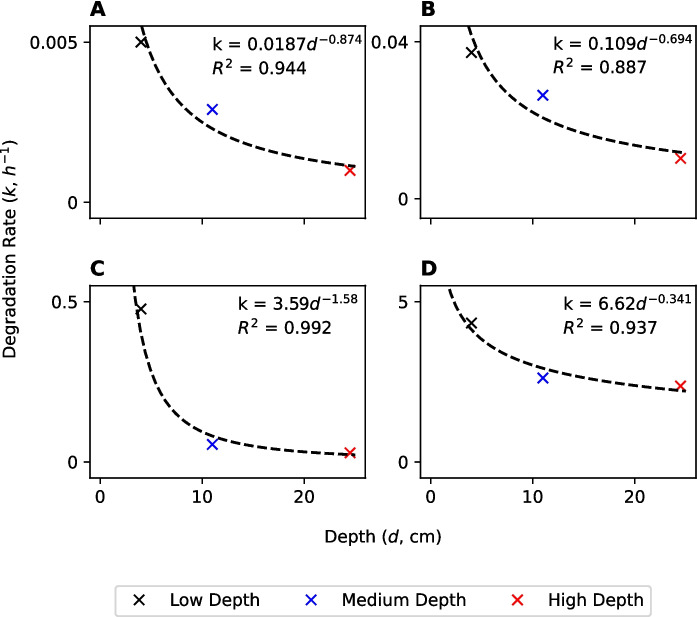
Relationships between transformation rate constants ($k$) and depth ($d$) for NH_4_^+^ (**A-B**) and LAS (**C-D**). Left panels (A and C) represent first-order rate coefficients and right panels (B and D) represent zero-order rate coefficients

Overall, the data clearly support the hypothesis that the rate constant for microbially-mediated chemical transformation is inversely proportional to the hydraulic radius (Fig. 4). The difference in FO rate constants between treatments and ZO rate constants between treatments (*k*) was also statistically significant ($p$ < 0.05) according to the bootstrapping procedure.

### Sediment Size Experiment

**Fig. 5:**
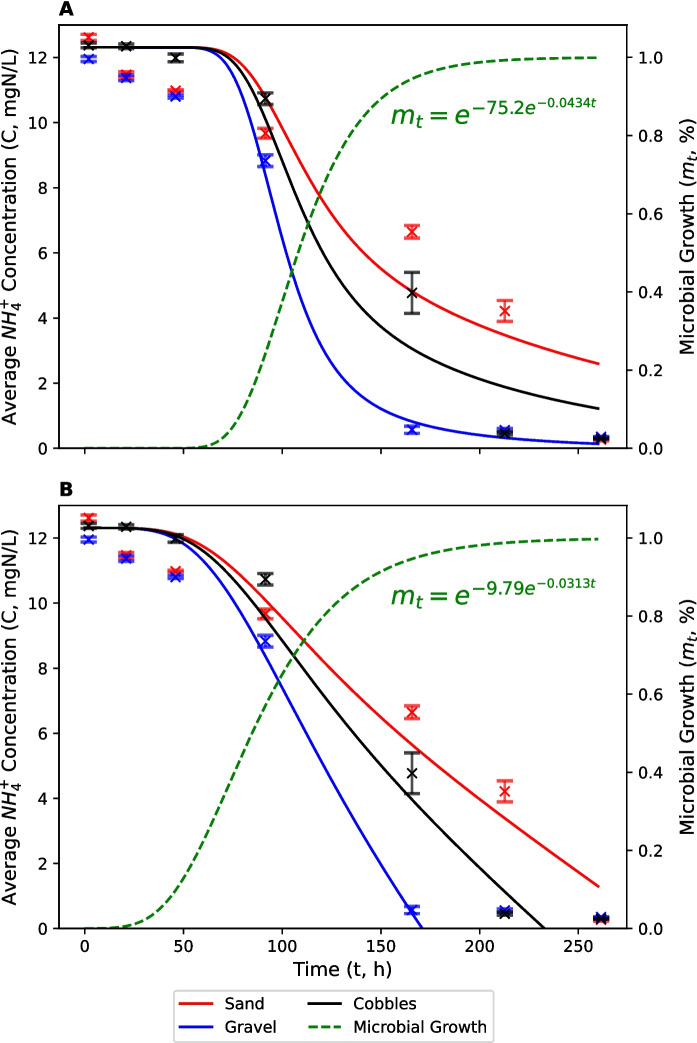
Average concentrations of NH_4_^+^-N in cobble (black), gravel (blue) and sand (red) treatments. Solid lines represent modified first-order (**A**) and zero-order (**B**) nitrification models which account for a lag period. The calibrated microbial growth curve (green/dashed) is also shown. Error bars represent standard errors

The average concentration of NH_4_^+^ across all treatments at the start of the sediment size experiment was 12.31 mg N L^−1^ (Fig. 5). This represents, again, a small but acceptable reduction in the nominal spiked concentration. NH_4_^+^ concentrations began to decrease appreciably after the same lag period as that observed in the channel geometry experiment (~ 2 d; 44 h). Again, microbial growth was predicted to be slightly quicker in the modified ZO model compared to the FO model. Overall, fitted nitrifier growth was slightly slower in the sediment size experiment, compared to the channel geometry experiment. This may have been due to differences in the microbial inoculum, river water quality and temperature. Environmental conditions and river water characteristics, such as temperature, pH, dissolved oxygen concentration and initial nitrifier population density, are all known to affect microbial growth (Antoniou et al., 1990; Cavari, 1977; Stenstrom & Poduska, 1980).

Nitrification was fastest in the gravel treatment, followed by the cobble treatment and then the sand treatment. NH_4_^+^ was < LOD by day 7 (164 h), day 9 (211 h) and day 11 (260 h) in each treatment, respectively. FO nitrification rate constants ($k$) were 1.73 × 10^–2^, 8.89 × 10^–3^ and 5.99 × 10^–3^ h^−1^, which represent half-lives of 40, 78 and 116 h, respectively (Table 3). ZO nitrification rate constants were 7.55 × 10^–2^, 5.32 × 10^–2^ and 4.25 × 10^–2^ mg N L^−1^ h^−1^, respectively. The difference in FO nitrification rate constants between treatments and ZO nitrification rate constants between treatments was statistically significantly ($p$ < 0.05). These results suggest that the rate constants for microbially-mediated transformations are not simply inversely proportional to bed sediment size, $D$. The relationship between $D$ and $k$ is also likely to be controlled by other factors, such as solute penetration to the bulk sediment volume, which will decrease in finer-grained material (Cook et al., 2020; O’Connor & Harvey, 2008). Optimal nitrification is, therefore, likely to be controlled by a combination of sediment surface area for microbial colonisation and solute exchange rates between the water column and the hyporheic zone, which will also be a function of sediment size distribution, packing and pressure gradients.

**Table 3:** Root-mean-square error (RMSE) and nitrification rate constants ($k$) for modified first-order and zero-order kinetics in the sediment size experiment

	First-Order			Zero-Order		
Sediment Size	$k$ (h^−1^)	RMSE (mg N L^−1^)	R^2^	$k$ (mg N L^−1^ h^−1^)	RMSE (mg N L^−1^)	R^2^
Sand	5.99 × 10^–3^	1.372	0.90	4.25 × 10^–2^	0.835	0.97
Gravel	1.73 × 10^–2^	0.706	0.99	7.55 × 10^–2^	0.723	0.99
Cobbles	8.89 × 10^–3^	0.925	0.97	5.32 × 10^–2^	0.616	0.98

**Fig. 6:**
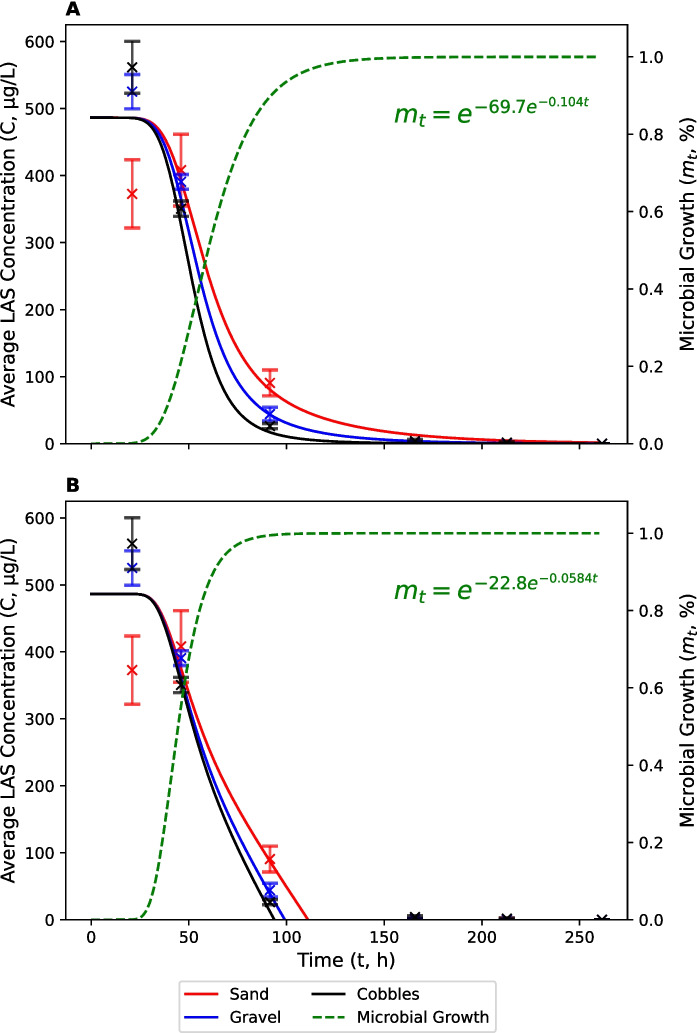
Average concentrations of LAS in cobble (black), gravel (blue) and sand (red) treatments. Solid lines represent modified first-order (**A**) and zero-order (**B**) biodegradation models which account for a lag period. The calibrated microbial growth curve (green/dashed) is also shown. Error bars represent standard errors

The average concentration of LAS at the start of the sediment size experiment was 330 μg L^−1^ which is much lower than the nominal spiked concentration (Fig. 6). Again, we believe that this was caused by a combination of matrix effects (35% reduction in spiked recovery) and the sorption of LAS to sediment (see above and Supplementary Materials). As in the channel geometry experiment, average LAS concentrations increased to 486 μg L^−1^ on day 1 (19 h) before decreasing to 383 μg L^−1^ on day 2 (44 h), which we ascribe to incomplete solubilisation of the spiked LAS after addition. Initial LAS concentrations were, therefore, excluded from the optimization of FO and ZO biodegradation models. The fitted lag period observed for LAS biodegradation in the sediment size experiment (~ 1 d) was shorter than the lag period observed for ammonia oxidation (~ 2 d). This could be due to the presence of a broader range of competent organisms in the original inoculum, compared with nitrifiers (Goodnow & Harrison, 1972; Koops et al., 2006). This lag period was also shorter than that observed in the channel geometry experiment. Again, this could be explained by differences in inoculum composition, environmental conditions and river water characteristics.

Average LAS concentrations in the cobble, gravel and sand treatments by day 3 (90 h) were 36, 44 and 91 μg L^−1^, respectively. Concentrations were < LOQ in all treatments by day 4 (164 h). Biodegradation was fastest in the cobble treatment, followed by the gravel treatment and then the sand treatment. FO degradation rate constants ($k$) were 4.08 × 10^–2^, 2.94 × 10^–2^ and 2.19 × 10^–2^, respectively (Table 4). These represent half-lives of 17.0, 23.5 and 31.6 h. ZO degradation rate constants ($k$) were 5.21, 4.92 and 4.39 h^−1^, respectively. Although there was a statistically significant difference in FO and ZO rate constants between treatments ($p$ < 0.05), the difference between treatments is small. Therefore, it remains difficult to draw conclusions about the relationships between $D$ and $k$.

**Table 4:** Root-mean-square error (RMSE) and biodegradation rate constants ($k$) for modified first-order and zero-order kinetics in the sediment size experiment

	First-Order			Zero-Order		
Sediment Size	$k$ (h^−1^)	RMSE (μg L^−1^)	R^2^	$k$ (μg L^−1^ h^−1^)	RMSE (μg L^−1^)	R^2^
Sand	2.19 × 10^–2^	33.9	0.97	4.39	51.3	0.97
Gravel	2.94 × 10^–2^	17.5	0.99	4.92	17.5	0.99
Cobbles	4.08 × 10^–2^	51.3	0.99	5.21	33.9	0.89

FO half-lives of NH_4_^+^ in the sediment size experiment (40–166 h) were all shorter than the half-life of the medium-depth treatment in the channel geometry experiment (239 h). These two treatments used the same volume of water but more gravel was used in the sediment size experiment (30 kg) than in the channel geometry experiment (10 kg). This means that there was an increased sediment surface area (SSA) for biofilm colonisation, which could explain the higher overall rate constant. Assuming spherical grains, 10 kg of gravel has a SSA of 7.58 m^2^, compared with 22.7 m^2^ for 30 kg gravel. Since each experiment was run at different times, this assertion should be treated with caution. FO half-lives for LAS in the sediment size experiment (17–32 h) were similar to the half-life of the medium-depth treatment in the channel geometry experiment (12.6 h), suggesting that additional surface area had less effect for LAS than for NH_4_^+^.

### Model Performance

The modified FO and ZO degradation curves, which account for a lag period in the development of competent bacteria, fit the data reasonably well (Figs. 2, 3 and 5, 6; Tables 1, 2, 3, 4). The average RMSE across all experiments and treatments was 0.780 mg N L^−1^ for nitrification and 38.0 μg L^−1^ for biodegradation. The coefficient of determination (R^2^) ranged from 0.86 to 0.99, with an average of 0.95. FO and ZO model performances were very similar, with an average RMSE of 0.890 mg N L^−1^ (FO) and 0.670 mg N L^−1^ (ZO) for nitrification, and 35.8 μg L^−1^ (FO) and 40.1 μg L^−1^ (ZO) for biodegradation. The average R^2^ was 0.92 (FO) and 0.96 (ZO) for nitrification, and 0.98 (FO) and 0.96 (ZO) for biodegradation.

Each model performed better than equivalent models without a lag period, where average RMSE was 1.40 mg N L^−1^ (FO) and 0.957 mg N L^−1^ (ZO) for nitrification, and 93.5 μg L^−1^ (FO) and 93.2 μg L^−1^ (ZO) for biodegradation. The average R^2^ for transformation models without a lag period were 0.89 (FO) and 0.93 (ZO) for nitrification, and 0.82 (FO) and 0.72 (ZO) for biodegradation. Despite an improvement in model performance with the inclusion of a lag period, fitted transformation rate constants ($k$) were similar to those when a lag period was not included. This suggests that reported values for $k$ are not artefacts of additional parameters in the calibration procedure.

### Environmental Relevance

Our experiments were deliberately abstract and designed to reveal the extent to which key system-specific controls can modify biotransformation rate constants. In the natural environment, the relationship between bed material characteristics and microbially-mediated chemical transformation will be complicated by sediment sorting, sediment stratification (Marzadri et al., 2024) and turbulence effects related to sediment size and bedform. Poorly-sorted bed material is predicted to behave in the same way as fine-bedded sediment because efficient grain packing reduces hydraulic conductivity but increases the sediment surface area available for biofilm colonisation (Cook et al., 2020; O’Connor & Harvey, 2008; Parker et al., 2018). Microbially-mediated transformation is likely to be more rapid in well-mixed (highly-turbulent) flow because the interaction between chemicals in solution and biofilms fixed to the bed and bank material is high. Therefore, transformations may occur more quickly in coarse-bedded streams because turbulence-induced mixing is likely to increase with sediment size (all other things being equal; Charlton, 2007). That said, biofilm development can be limited in some turbulent flows because high shear stress can detach portions of the biofilm (sloughing) (Lau & Liu, 1993; Stoodley et al., 2001). The presence of bedforms (e.g. dunes, bars and ripples) in natural river systems induces pressure gradients which drive advective water flow in, through and out of bed sediments (Cook et al., 2020; Li et al., 2015). This mechanism is known as ‘pumping’ (Wörman et al., 2002). The increased turnover of water and chemicals in solution in these biologically-active hyporheic zones is predicted to increase microbial transformation of chemicals overall (Li et al., 2015; Posselt et al., 2020). Therefore, in addition to further research on the relationship between bed material characteristics and microbially-mediated chemical processing, research is also needed to determine how this understanding translates to the natural environment.

Across both experiments, ZO degradation rate coefficients for NH_4_^+^ (0.01–0.08 mg N L^−1^ h^−1^) were similar in magnitude to a field-derived ZO coefficient for a river channel in Laos (0.06 mg N L^−1^ h^−1^; Whelan et al., 2007). However, FO half-lives for NH_4_^+^ (40–695 h) were much longer than those typically reported from field data for rivers. For example, the apparent half-life for NH_4_^+^ reported for a shallow UK stream was 2.4 h (Fox et al., 2000). Similarly, a value of 2.97 h was reported for a shallow river in the Philippines (McAvoy et al., 2003). Shorter half-lives in the field probably arise as a consequence of long-term exposure to chemicals (and associated microbial acclimation) as well as from an expected higher complexity in natural biofilms which could lead to higher and more resilient microbial competence and, thus, shorter half-lives (Guhl & Steber, 2006; Kowalczyk et al., 2015). This may be, in part, an indirect consequence of the influence of phototrophs in the field, which have been shown to enhance photostable fungicide degradation in laboratory studies (Southwell et al., 2020). The half-lives reported here were also longer than in some other laboratory experiments. For example, Finnegan et al. (2009) reported a first-order half-life for NH_4_^+^ of 7.8 h in a linear channel cascade after acclimation and Le et al. (2019) reported a half-life of 6.42 h in a bench-scale laboratory mesocosm experiment. These differences may be due to differences in inocula, temperature and acclimation as well as channel morphology.

Across both experiments, FO half-lives for LAS (1.45—31.6 h) were similar in magnitude to those reported in rivers and in other laboratory simulation experiments. For example, half-lives in other lab-based artificial river systems of 2.8 and 7.1 h have been reported by Boeije et al. (2000) and Finnegan et al. (2009), respectively. In the field, half-lives between 0.9 and 36 h have been reported for rivers in the UK (Fox et al., 2000), USA (Morrall et al., 2004; Rapaport & Eckhoff, 1990), Italy (Facchi et al., 2007; Whelan et al., 1999), Japan (Takada et al., 1994), Laos (Whelan et al., 2007) and the Philippines (McAvoy et al., 2003). These comparisons suggest that the rate constants reported here are relevant to real rivers, despite our deliberately abstract experimental design.

It should be noted that the reported values of $k$ represent removal from all potential loss mechanisms, including microbial transformation, volatilisation and sorption to sediment (Mackay, 1991). In the case of LAS, volatilisation is likely to be negligible because LAS has a very low Henry’s Law constant (6.35 × 10^–3^ Pa m^3^ mol^−1^). For NH_4_^+^, volatilisation is likely to be more important because it is in equilibrium with unionised (free) ammonia (NH_3_), which is volatile. However, < 6% of the ammoniacal nitrogen was calculated to be in the form of NH_3_ in our experimental system. This calculation is based on:

5 $${f}_{N{H}_{3}/N{H}_{4}^{+}} = \frac{1}{1+{10}^{(\mathrm{pKa}-\mathrm{pH})}}$$

where pKa is 9.55 at 15.5 °C (Emerson et al., 1975; Whelan et al., 2010). The average temperature and pH measured before each sample was collected were 15.5 °C and pH 8.28.

The approximate magnitude of the first-order volatilization rate constant for losses of total ammoniacal N (*k*_*v*_, h^−1^) was calculated from:

6 $${k}_{v}=\frac{f.{k}_{WA}}{d}$$

where *f* is the fraction of total ammoniacal N in unionized form (i.e. 0.051), *d* is the water depth (m) and *k*_*WA*_ (m h^−1^) is the mass transfer coefficient for water to air transport, which is calculated from two-film resistance theory (Liss & Slater, 1974):

7 $${k}_{WA}={\left(\frac{1}{{k}_{w}}+\frac{1}{{k}_{A}.{K}_{AW}}\right)}^{-1}$$

where *k*_*W*_ and *k*_*A*_ are the partial mass transfer coefficients on the water side and air side of the water–air interface, respectively and *K*_*AW*_ is the air–water partition coefficient (i.e. the dimensionless Henry’s Law constant). We employed parameters for volatilization derived experimentally by Whelan et al. (2010) of *k*_*W*_ = 0.00189 m h^−1^, *k*_*A*_ = 0.189 m h^−1^ and *K*_*AW*_ = 0.003 which resulted in values for *k*_*v*_ of 0.0003 and 0.0001 h^−1^ for the shallowest and deepest treatments, respectively. These values represent approximately 12% and 10%, respectively, of the apparent overall rate constant for loss of ammoniacal N reported in Table 1, indicating that volatilization is likely to have made a relatively minor contribution to losses in our experiments. We also considered sorption to sediment. While some initial NH_4_^+^ loss through sorption is possible at the start of the experiment, net losses from the water column are expected to be minimal once the sediment and overlying water approach thermodynamic equilibrium.

DO concentrations were also above the thresholds that limit aerobic nitrification and biodegradation (4 mg L^−1^ at most; Krueger et al., 1998; León et al., 2006; Schübl et al., 2021; Stenstrom & Poduska, 1980). There was little difference in temperature, pH or DO concentrations between treatments, systems, experiments or over time. Although some net water column losses of both LAS and NH_4_^+^ due to sorption are possible (especially at the start of the experiment; see Supplementary Materials), these losses are expected to be very low once the sediment and overlying water approach thermodynamic equilibrium (Boeije et al., 2000; Finnegan et al., 2009; Larson, 1990; Newbold et al., 1983). Net sorption to suspended sediment is also predicted to be low once equilibrium partitioning is established.

The co-presence of LAS and NH_4_^+^ in our experiments could potentially have produced interactions which may have influenced the transformation rates, although both contaminants are expected to co-occur in rivers downstream of domestic wastewater effluent sources which implies that any such interactions will be field-relevant. Possible interactions include: (1) toxicity of one pollutant on the micro-organisms which mediate transformation of the other; (2) stimulatory effects and (3) effects of one pollutant on the availability of the other to competent micro-organisms (e.g. due to sorption). High concentrations of LAS have been shown to inhibit nitrification (Brandt et al., 2001; Teng et al., 2024; Wu et al., 2020). However, most studies report inhibitory concentrations which are much higher than those which are typically observed in treated wastewater or river water. Wu et al. (2020) examined NH_4_^+^ depletion at LAS concentrations of 10 mg L^−1^ and 30 mg L^−1^. Brandt et al. (2001) observed only a minor reduction in ammonia oxidation at a LAS concentration of 3 mg L^−1^. This is almost twice the maximum SDBS concentration used in our experiments (1.55 mg L^−1^). Similarly, Teng et al. (2024) reported only negligible differences in aerobic NH_4_^+^ depletion over a range of SDBS concentrations up to 2.5 mg L^−1^. Therefore, reported inhibition thresholds for nitrification are typically much higher than those used in our experiments. This conclusion is also supported by studies in soil. For example, Madsen et al. (1998) reported that LAS did not cause observable effects on soil nitrification in soils amended with aqueous extracts of LAS-spiked sewage sludge at concentrations up to 19 g kg sludge^−1^. The potential for ammonia to have had any toxic effect on the heterotrophic micro-organisms which mediate LAS biodegradation is likely to have been minimal, in part because concentrations of unionised ammonia in our experiments are likely to be < 0.76 mg N L^−1^. However, it is possible that NH_4_^+^ may have had some stimulatory effect on heterotroph growth and activity. Any such effects will have been relatively uniform across treatments and over time because most heterotrophic bacteria can utilize both NH_4_^+^ and NO_3_^−^ as sources of mineral N, although they typically have a preference for NH_4_^+^ (Stanier et al., 1976). Competitive ion exchange effects are unlikely because SDBS forms an anion in solution whereas the majority of ammoniacal N is cationic.

In summary, the principal mechanism influencing the effect of hydraulic radius on the overall rate constant for both SDBS and NH_4_^+^ is the advective transfer of the contaminant from the water-column to the sediment (and, for volatilization, from the water-column to the air–water interface), once microbial colonization has reached steady state. In other words:

8 $$k=\frac{{k}_{WS}}{R}$$

where *k* is the overall first order rate constant (h^−1^), *R* is the hydraulic radius (m) and *k*_*ws*_ is the mass transfer coefficient (m h^−1^) between the water column and the bed (sometimes referred to as the uptake velocity; Liu et al., 2022; Maavara et al., 2017). After a steady state microbial community is established changes to microbial community size or activity should play no further role in controlling the temporal dynamics of pollutant concentrations in the experimental system.

### Implications for Exposure Modelling

Our results have some important implications for chemical exposure modelling and associated environmental risk assessment. Most current in-stream exposure models, such as SIMCAT (Crabtree et al., 2009), iSTREEM (Kapo et al., 2016) and GREAT-ER (Feijtel et al., 1997) employ a single first-order rate constant ($k$) for all reaches in a channel network. However, this assumption will be increasingly inappropriate at the large catchment scale because $k$ is likely to be modified by systematic and significant changes to channel shape and size and bed material characteristics. Perennial rivers tend to systematically widen and deepen with distance downstream as discharge accrues (Leopold & Maddock, 1953). In parallel, bed material typically gets finer, often with an abrupt gravel-sand transition, in distal reaches (Charlton, 2007). These features will drive a systematic decrease in *k* with distance downstream with significant implications for predicted local exposures and for the export of pollutants from freshwater catchments to the marine system (e.g. Alexander et al., 2000). Thus, the inclusion of geomorphology in chemical exposure models could potentially improve their performance. Further research is needed to determine the relative importance of geomorphology on the net effects of microbially-mediated transformations in the field over large temporal and spatial scales (e.g. over timeframes of a year or more and between low and high-order streams; Honti et al., 2018). Our results also provide support for the maintenance of natural river channel morphology and the use of river restoration as a method to improve chemical water quality. River restoration typically involves the reintroduction of meander bends and natural bed substrates to replace straightened and concrete-lined channels (Wohl et al., 2015). This is likely to diversify the channel habitat, increase the surface area available for biofilm colonisation on the banks and channel bed, enhance hyporheic exchange and increase net reach-scale transformation rate constants over space and time.

## Conclusions

Two experiments were conducted to investigate the influence of channel geometry and bed sediment particle size on microbially-mediated transformations of wastewater chemicals in rivers. We observed that microbially-mediated transformation rate coefficients were inversely proportional to depth, which supported our initial hypothesis. This morphological control on effective rate coefficients has been previously recognised for nitrification and denitrification, but its role in organic chemical behaviour is less well established. The effects of sediment size on chemical transformation rates were more complex. Our initial hypothesis was that transformation rate coefficients would be inversely proportional to sediment size, due to increased surface area per unit sediment mass for periphyton colonisation. However, this was not supported by the data. This is probably due to the combined effects of particle size and packing on sediment permeability and associated hyporheic exchange, as well as on biofilm colonisation.

## Supplementary Information

Below is the link to the electronic supplementary material.

Supplementary file1 The following information is provided as Supplementary Material: (1) LAS characteristics, (2) LC-MS/MS conditions for the analysis of LAS (e.g. column type, mobile phase and MS source conditions), (3) LAS LC-MS/MS method development and validation, (4) the calculation of matrix effects, and (5) estimates of LAS sorption to sediment. (DOCX 606 kb)

## Data Availability

Data will be made available on request.
