# Supplementary material for: Effects of Channel Characteristics on Wastewater Chemical Transformation in Rivers
Source: Water Air Soil Pollut. 2026 Feb 5;237(9):507. doi: 10.1007/s11270-026-09211-y (PMC12876528; doi:10.1007/s11270-026-09211-y)
Supplement: Supplementary file 1 — Supplementary file1 The following information is provided as Supplementary Material: (1) LAS characteristics, (2) LC-MS/MS conditions for the analysis of LAS (e.g. column type, mobile phase and MS source conditions), (3) LAS LC-MS/MS method development and validation, (4) the calculation of matrix effects, and (5) estimates of LAS sorption to sediment. (DOCX 606 kb) [file 11270_2026_9211_MOESM1_ESM.docx]

Supporting Material For

**Effects of Channel Characteristics on Wastewater Chemical Transformation in Rivers**

Robert A. Newbould^a^*^#^, D. Mark Powell^a^, Juliet Hodges^b^, Alexandre Teixeira^b^, Michael J. Whelan^a^

^a^ School of Geography, Geology and the Environment, University of Leicester, Leicester, LE1 7RH, UK

^b^ Unilever Safety, Environmental and Regulatory Science (SERS), Colworth Science Park, Sharnbrook, MK44 1LQ, UK

* Corresponding Author: [ran14@leicester.ac.uk](mailto:ran14@leicester.ac.uk)

^#^ Present Address: Yorkshire Water Services, Bradford, BD6 2SZ, UK

([robert.newbould@yorkshirewater.co.uk](mailto:robert.newbould@yorkshirewater.co.uk))

**Supplementary Information: LAS Characteristics**

**
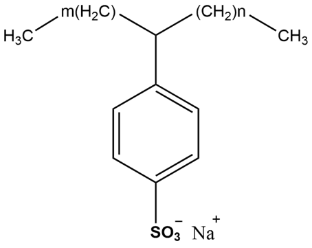
**

**Figure S1 –** General chemical structure of linear alkylbenzene sulfonate, with the typical technical mixture composed of analogues with a linear alkyl chain of C_10_ to C_13_

**Table S1 –** Analogue distribution of the material used in this study (sodium dodecylbenzene sulphonate from Sigma-Aldrich) as determined by direct infusion mass spectrometry

| **LAS Analogue** | **C_10_** | **C_11_** | **C_12_** | **C_13_** |
| --- | --- | --- | --- | --- |
| Distribution | 17% | 44% | 31% | 4% |

**Supplementary Information: LC-MS/MS Conditions for Analyses of LAS**

**Instrument:** Agilent 1290 series LC-MS/MS System with 6495 triple quadrupole mass spectrometer

**Clean Up Column:** Thermo Hypercarb 3 μmm 2.1 x 10 mm guard column (placed in between pump outlet and injector flow)

**Analytical Column:** XBridge BEH shield RP18 2.5 μm, 2.1 x 50 mm column XP + equivalent 5mm guard-column

**Column Temperature:** 30°C

**Injection Volume:** 3 μL

**Flow Rate:** 0.7 mL/min

**Mobile Phase:**Water (95%)/MeoH (5%) + 0.1% formic acid (A) / Methanol (95%)/Water (5%) + 0.1% formic acid (B)

**Gradient:**

| **min** | 0 | 0.7 | 1.0 | 2.0 | 6.3 | 6.4 | 8.0 |
| --- | --- | --- | --- | --- | --- | --- | --- |
| **%A** | 40 | 40 | 15 | 0 | 0 | 40 | 40 |

**MS Source Conditions:**

- Mode: ESI negative
- Source Gas Temperature: 230°C
- Sheath Gas Temperature: 230°C
- Capillary Voltage: 4000v
- Nozzle Voltage: 500v
- High Pressure RF: 210v
- Low Pressure RF: 120v

**MS/MS Conditions:**

| **Compound** | **Precursor Ion (m/z)** | **Product Ion (m/z)** | **Collision Energy (eV)** |
| --- | --- | --- | --- |
| C_10_ LAS | 297.2 | 183 | 38 |
| C_11_ LAS | 311.2 | 183 |  |
| C_12_ LAS | 325.2 | 183 |  |
| C_13_ LAS | 339.2 | 183 |  |

**Supplementary Information:** **LAS LC-MS/MS Method Development and Validation**

The extracted ion chromatograms for each LAS analogue were obtained using the LC-MS/MS conditions stated above (Figure S2). The unresolved peaks within each analogue represent the different attachment positions of the benzene sulfonate ring (positional isomers). For the purposes of this study, no distinction between isomers was made and these were all integrated and quantified as a single peak.

Calibration curves were generated for each LAS analogue (Figure S3). Each calibration standard was run in duplicate and triplicate at the limit of quantification (LOQ), with a 1/x fit. An analytical run was considered valid if R^2^ > 0.99 and the back calculated concentration of each standard was within 80 to 120% of the nominal value. Quality control standards, prepared separately at three different concentrations covering the linear range, were injected throughout each run. The acceptance criteria were a coefficient of variation (CV) of less than 20% and a calculated concentration within 20% of the nominal value.

Due to the widespread use of LAS, high background levels were observed and the LOQ for each LAS analogue was driven by the blank levels (Table S2). In this case, the LOQ was determined as the lowest concentration that was consistently twice the average response in ultrapure water, with a CV < 20% (n = 3).


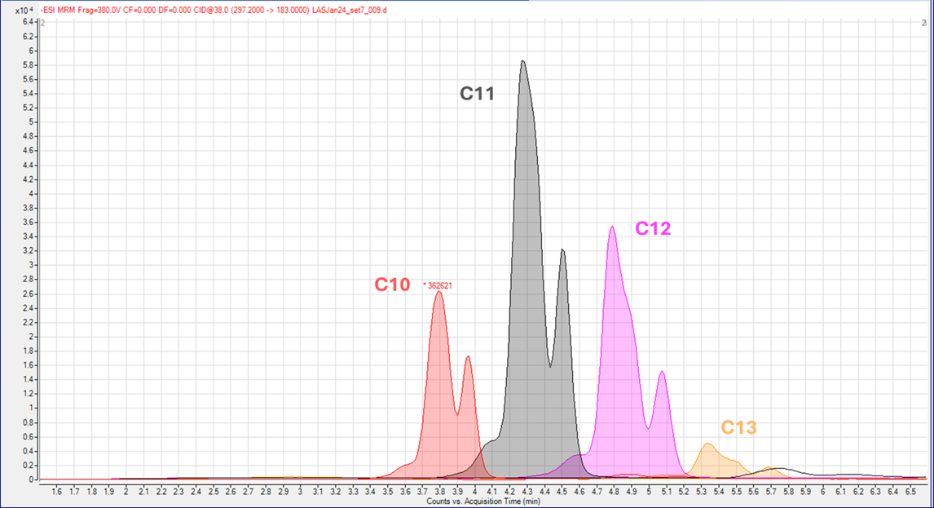


**Figure S2 –** Overlay of extracted ion chromatograms for C_10_ to C_13_ LAS


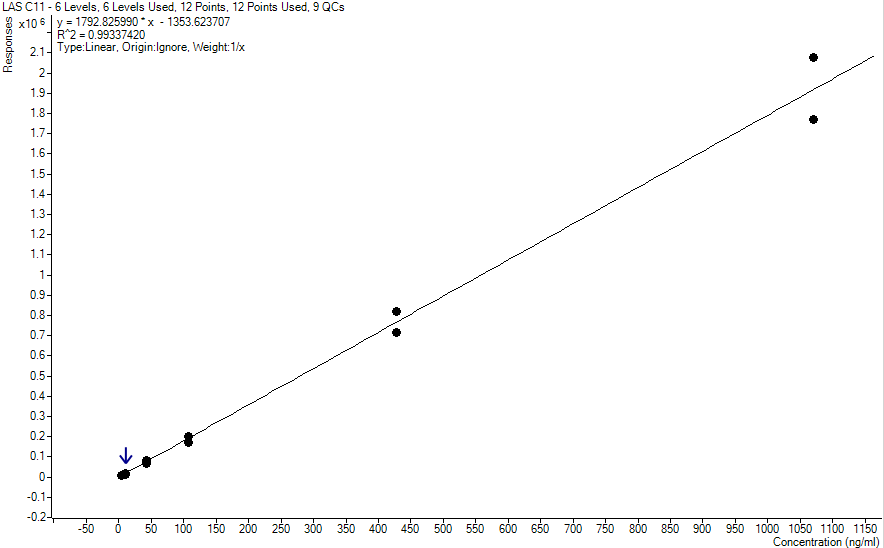

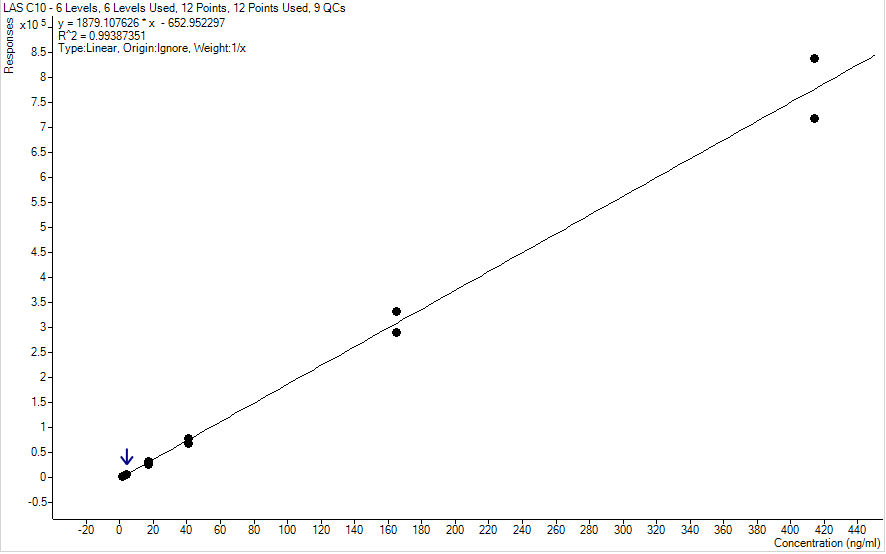


C_11_ LAS

C_10_ LAS


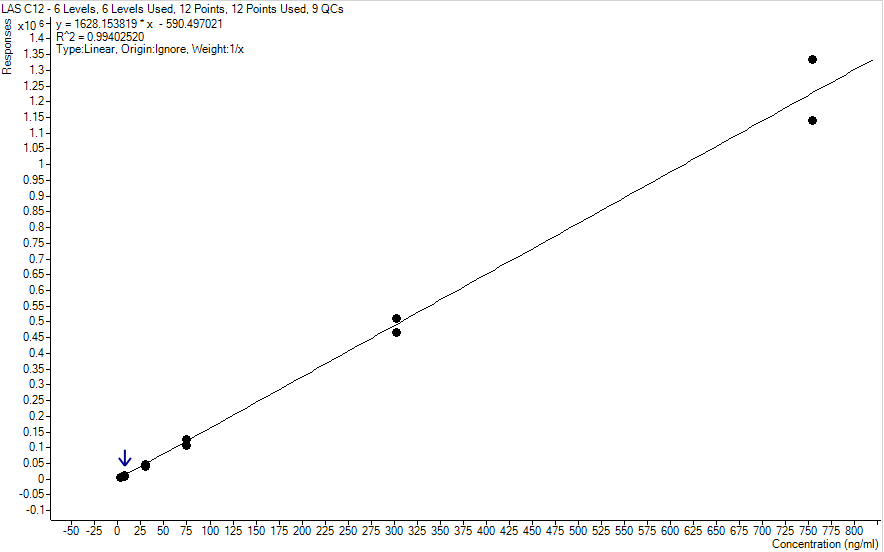

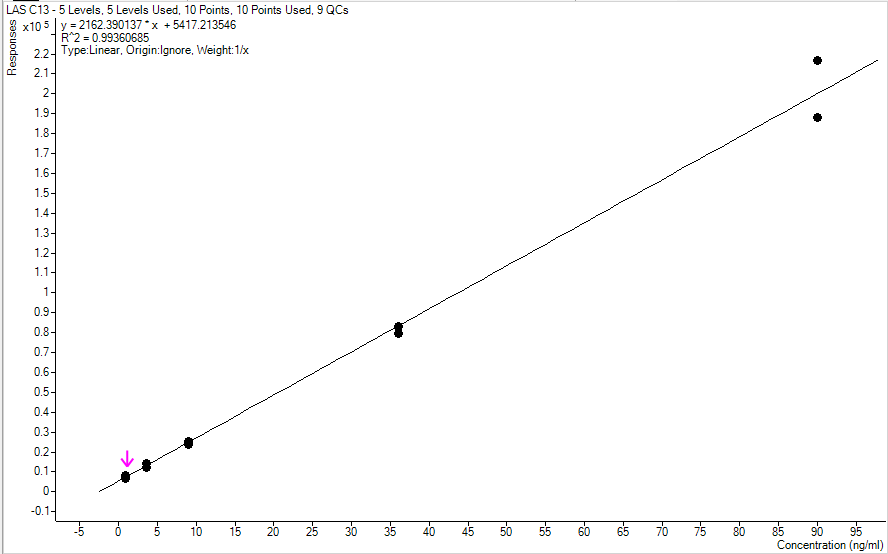


C_13_ LAS

C_12_ LAS

**Figure S3 –** Calibration curves for each LAS analogue from the sediment size experiment

**Table S2 –** Limits of quantification (LOQ) for each LAS analogue (ng mL^-1^)

| **C_10_** | **C_11_** | **C_12_** | **C_13_** |
| --- | --- | --- | --- |
| 1.7 | 4.4 | 3.1 | 0.925 |

**Supplementary Information: Matrix Effects**

In both the channel geometry experiment and sediment size experiment, matrix effects were believed to cause reductions in spiked recoveries of LAS. Matrix effects refer to the suppression or enhancement of analytical signals caused by co-eluting substances from the sample matrix – in this case, river water. To determine the extent of matrix effects, a quality control standard with 250 μg L^-1^ each of C_10_-C_13_ LAS in river water was prepared. Table S3 details the amount of LAS detected in three replicates of this standard, alongside percent recovery. The average recovery rate was 65%. This suggests a 35% reduction in spiked concentrations of LAS. Reproducibility between replicates was good, with a consistent CV of 4% for each LAS homologue. To improve recovery, further method development is needed, which was limited by the available time and resources in this study. For example, adding an internal standard with similar structure and retention time to LAS (ideally isotope labelled versions of each LAS analogue) could improve accuracy. This was deemed impractical due to the high number of samples analysed.

**Table S3** **–** Amount of LAS (per species) detected from the 250 μg L^-1^ LAS quality control standard, along with percent recovery. CV is the coefficient of variation between replicates

|  | **LAS Species (ug L^-1^)** | | | |
| --- | --- | --- | --- | --- |
| **Replicate** | **C_10_** | **C_11_** | **C_12_** | **C_13_** |
| 1 | 184 | 171 | 153 | 135 |
| 2 | 182 | 169 | 156 | 156 |
| 3 | 195 | 181 | 164 | 164 |
| Average | 187 | 174 | 158 | 136 |
| CV | 4% | 4% | 4% | 4% |
| Recovery | 75% | 69% | 63% | 54% |

**Supplementary Information: Sorption of LAS to Sediment**

In addition to matrix effects, the sorption of LAS to sediment is likely to have contributed to a reduction in initial concentrations. In most rivers and streams, LAS sorption is not typically considered a significant removal mechanism due to the fact that wastewater emissions are approximately steady state which means that thermodynamic equilibrium partitioning between bed sediment and the overlying water column can be assumed, with no substantial net removal of LAS from the water column to the sediment^1–3^. However, in this experiment, the commercial sediment did not initially contain any LAS, making some net sorption plausible. Sorption equilibrium typically occurs rapidly (within 3 h^3^). Thereafter, further net sorption is unlikely. In fact, microbial transformation in the water column may lead to LAS desorption to maintain equilibrium, thereby slightly offsetting 'actual' transformation rates. The measurement of parallel processes (such as sorption and desorption) is an inherent flaw of this experiment and other simulations tests, such as the OECD 308 Test^4^.

In order to estimate the sorbed fraction of LAS, we considered a simple mass balance for LAS, resulting in^5^:

$C_{w}=\frac{M}{V_{w}+ V_{s}k_{d}}$ (S1)

where $C_{w}$ is the concentration of LAS in water (mg L^-1^), $M$ is mass of LAS added (mg), $V_{w}$ is the volume of water (L), $V_{s}$ is the volume of sediment (kg) and $k_{d}$is the adsorption coefficient (L kg^-1^). $k_{d}$ is the ratio of $C_{s}$ (the concentration of LAS in sediment, mg kg^-1^) to $C_{w}$. Table S4 summarises the parameters used in Equation S1 (except $k_{d}$) for each treatment in the channel geometry and sediment size experiments. Note that $M$ was adjusted depending on the extent of matrix effects (see above).

**Table S4** **–** Input parameters to estimate the sorbed faction of LAS in the channel geometry and sediment size experiments. $M$ is the mass of LAS added (mg), $V_{w}$ is the volume of water (L) and $V_{s}$ is the volume sediment (kg)

|  | **Depth Treatment** | | | **Sediment Treatment** | | |
| --- | --- | --- | --- | --- | --- | --- |
| **Parameter** | **Low** | **Medium** | **High** | **Sand** | **Gravel** | **Cobbles** |
| $\boldsymbol{M}$ | 20.16 | 50.39 | 100.8 | 50.39 | 50.39 | 50.39 |
| $\boldsymbol{V}_{\boldsymbol{w}}$ | 20 | 50 | 100 | 50 | 50 | 50 |
| $\boldsymbol{V}_{\boldsymbol{s}}$ | 10 | 10 | 10 | 23 | 30 | 35 |

The adsorption coefficient, $k_{d}$, was calculated with:

$k_{d}= k_{oc} f_{oc}$ (S2)

where $k_{oc}$ is the organic carbon-water partition coefficient (L kg^-1^) and $f_{oc}$ is the fraction of organic carbon. $k_{oc}$ can be experimentally measured, for example with the OECD 121 Test, or estimated, for example from the octanol-water partition coefficient ($k_{ow}$). Note that for surfactants, $k_{ow}$ is experimentally difficult to determine. Therefore, $k_{oc}$ values derived from $k_{ow}$ may not adequately describe sorption and desorption. A range of $\log k_{oc}$ values between 1.57 and 4.93 have been measured or estimated for LAS (see Tables S5 and S6). The fraction of organic carbon, $f_{oc}$, in the commercial sediment used for this was study not measured. However, $f_{oc}$ was predicted to be low (< 1%) compared to soil^6^, which has a much higher organic carbon content. To account for the variability in potential $k_{oc}$ and $f_{oc}$ values, a range of these values were used to estimate the sorbed fraction of LAS (Figure S4). Both experimentally derived and estimated values of $k_{oc}$ were used. Figure S4 indicates that reductions in the spiked concentrations of LAS observed at the start of the channel geometry and sediment size experiments could plausibly be attributed to LAS sorption, as the observed concentrations fall within the predicted model range.

**Table S5 –** Experimentally derived log K_oc_ values

| **log K_oc_** | **Notes** | **Source** |
| --- | --- | --- |
| 3.40 | Calculated as C_11.6_ (average LAS chain length) | Feijtel et al. (1999)^7^  HERA (2013)^8^ |
| 3.92-4.93 | This range of log K_oc_ values represents the association between C_10_ or C_12_ LAS and various dissolved humic substances | Traina et al. (1996)^9^ |

**Table S6 –** Estimated log K_oc_ values

| **log K_oc_** | **Method** | **Source** |
| --- | --- | --- |
| 1.57-2.93 | Estimated from the Karickhoff relationship (K_oc_ = 0.41 K_ow_)^10^ using a range of log K_ow_ values | log K_ow_ = 1.96 ^11^  log K_ow_ = 3.00 ^12^  log K_ow_ = 3.35 ^8^ |
| 2.01-2.78 | Estimated using EPI Suite KOCWIN with the same range of log K_ow_ values as above | EPI Suite^12^ |
| 2.04-2.44 | Calculated for C_12_ LAS from water solubility and log K_ow_ | Lyman et al. (1990)^13^  HERA (2013)^8^ |
| 4.09 | Estimated from the molecular connectivity index using EPI Suite KOCWIN | EPI Suite^12^ |


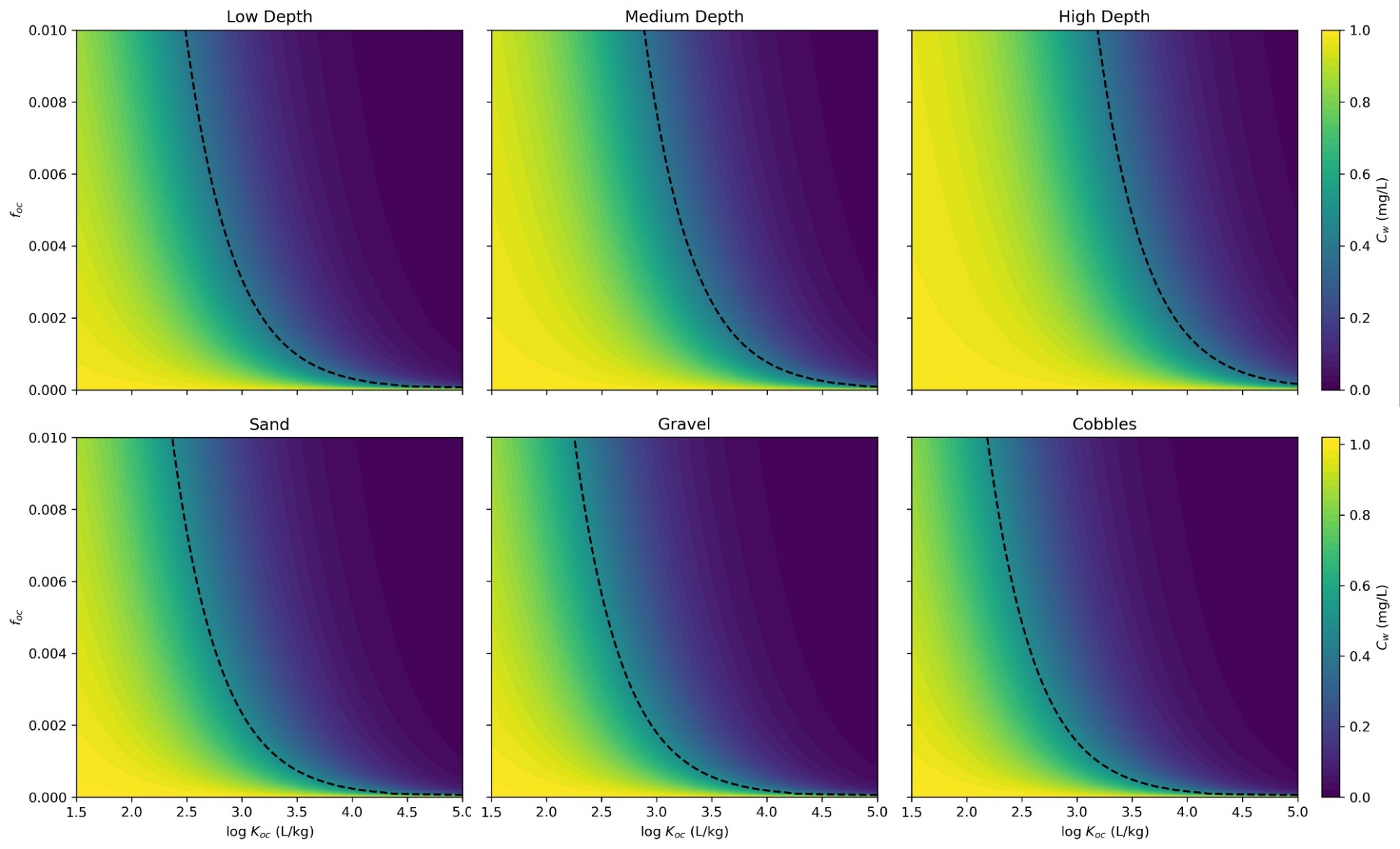


**Figure S4** **–** The concentration of LAS in water ($C_{w}$) as a function of log K_oc_ and the fraction of organic carbon ($f_{oc}$). The dashed line represents the average peak concentration of LAS measured across treatments in each experiment (391 μg L^-1^ in the channel geometry experiment and 486 μg L^-1^ in the sediment size experiment).

References

(1) Boeije, G. Incorporation of Biofilm Activity in River Biodegradation Modeling: A Case Study for Linear Alkylbenzene Sulphonate (LAS). *Water Res.* **2000**, *34* (5), 1479–1486. https://doi.org/10.1016/S0043-1354(99)00279-1.

(2) Finnegan, C. J.; Van Egmond, R. A.; Price, O. R.; Whelan, M. J. Continuous-Flow Laboratory Simulation of Stream Water Quality Changes Downstream of an Untreated Wastewater Discharge. *Water Res.* **2009**, *43* (7), 1993–2001. https://doi.org/10.1016/j.watres.2009.01.031.

(3) Larson, R. J. Structure-Activity Relationships for Biodegradation of Linear Alkylbenzenesulfonates. *Environ. Sci. Technol.* **1990**, *24* (8), 1241–1246. https://doi.org/10.1021/es00078a012.

(4) ter Horst, M. M. S.; Koelmans, A. A. Analyzing the Limitations and the Applicability Domain of Water–Sediment Transformation Tests like OECD 308. *Environ. Sci. Technol.* **2016**, *50* (19), 10335–10342. https://doi.org/10.1021/acs.est.6b02906.

(5) Mackay, D. *Multimedia Environmental Models: The Fugacity Approach*; Lewis Publishers: Chelsea, MI, 1991.

(6) ECETOC. *Soil and Sediment Risk Assessment of Organic Chemicals. Technical Report No. 92*; 2004. https://www.ecetoc.org/wp-content/uploads/2014/08/ECETOC-TR-092.pdf (accessed 2024-06-26).

(7) Feijtel, T. C. J.; Struijs, J.; Matthijs, E. Exposure Modeling of Detergent Surfactants—Prediction of 90th-Percentile Concentrations in the Netherlands. *Environ. Toxicol. Chem.* **1999**, *18* (11), 2645–2652. https://doi.org/10.1002/etc.5620181134.

(8) HERA. *Linear Alkylbenzene Sulphonate*; 2013. https://www.heraproject.com/files/HERA-LAS%20revised%20April%202013%20Final1.pdf (accessed 2024-06-26).

(9) Traina, S. J.; McAvoy, D. C.; Versteeg, D. J. Association of Linear Alkylbenzenesulfonates with Dissolved Humic Substances and Its Effect on Bioavailability. *Environ. Sci. Technol.* **1996**, *30* (4), 1300–1309. https://doi.org/10.1021/es950512r.

(10)Karickhoff, S. W. Semi-Empirical Estimation of Sorption of Hydrophobic Pollutants on Natural Sediments and Soils. *Chemosphere* **1981**, *10* (8), 833–846. https://doi.org/10.1016/0045-6535(81)90083-7.

(11) Hand, V. C.; Williams, G. K. Structure-Activity Relationships for Sorption of Linear Alkylbenzenesulfonates. *Environ. Sci. Technol.* **1987**, *21* (4), 370–373. https://doi.org/10.1021/es00158a006.

(12)US EPA. Estimation Programs Interface Suite^TM^ for Microsoft® Windows, v 4.11, 2012.

(13)Lyman, W. J.; Reehl, W. F. *Handbook of Chemical Property Estimation Methods: Environmental Behavior of Organic Compounds*; American Chemical Society, 1990.
